# Disorder-Specific and Shared Brain Abnormalities During Vigilance in Autism and Obsessive-Compulsive Disorder

**DOI:** 10.1016/j.bpsc.2016.12.005

**Published:** 2017-11

**Authors:** Christina O. Carlisi, Luke Norman, Clodagh M. Murphy, Anastasia Christakou, Kaylita Chantiluke, Vincent Giampietro, Andrew Simmons, Michael Brammer, Declan G. Murphy, MRC AIMS Consortium, David Mataix-Cols, Katya Rubia

**Affiliations:** a Department of Child and Adolescent Psychiatry, Sackler Institute for Translational Neurodevelopmental Sciences, London; b Department of Forensic and Neurodevelopmental Sciences, Sackler Institute for Translational Neurodevelopmental Sciences, Psychology and Neuroscience, King’s College, London; c Department of Neuroimaging, Psychology and Neuroscience, King’s College, London; d National Institute for Health Research Biomedical Research Centre for Mental Health at South London and Maudsley NHS Foundation Trust and Institute of Psychiatry, Psychology and Neuroscience, King’s College, London; e Behavioural Genetics Clinic, Adult Autism Service, Behavioural and Developmental Psychiatry Clinical Academic Group, South London and Maudsley NHS Foundation Trust, London; f Centre for Integrative Neuroscience and Neurodynamics, School of Psychology and Clinical Language Sciences, University of Reading, Reading, United Kingdom; g Department of Neurobiology, Care Sciences and Society (AS), Center for Alzheimer Research, Division of Clinical Geriatrics, Stockholm, Sweden; h Department of Clinical Neuroscience(DM-C), Centre for Psychiatry Research, Karolinska Institutet, Stockholm, Sweden

**Keywords:** Adolescence, ASD, Attention, fMRI, OCD, Vigilance

## Abstract

**Background:**

Autism spectrum disorder (ASD) and obsessive-compulsive disorder (OCD) are often comorbid and share similarities across some cognitive phenotypes, including certain aspects of attention. However, no functional magnetic resonance imaging studies have compared the underlying neural mechanisms contributing to these shared phenotypes.

**Methods:**

Age- and IQ-matched boys (11–17 years old) with ASD (*n* = 20), boys with OCD (*n* = 20), and healthy control boys (*n* = 20) performed a parametrically modulated psychomotor vigilance functional magnetic resonance imaging task. Brain activation and performance were compared among adolescents with OCD, adolescents with ASD, and control adolescents.

**Results:**

Whereas boys with ASD and OCD were not impaired on task performance, there was a significant group by attention load interaction in several brain regions. With increasing attention load, left inferior frontal cortex/insula and left inferior parietal lobe/pre/post-central gyrus were progressively less activated in boys with OCD relative to the other two groups. In addition, boys with OCD showed progressively increased activation with increasing attention load in rostromedial prefrontal/anterior cingulate cortex relative to boys with ASD and control boys. Shared neurofunctional abnormalities between boys with ASD and boys with OCD included increased activation with increasing attention load in cerebellum and occipital regions, possibly reflecting increased default mode network activation.

**Conclusions:**

This first functional magnetic resonance imaging study to compare boys with ASD and OCD showed shared abnormalities in posterior cerebellar–occipital brain regions. However, boys with OCD showed a disorder-specific pattern of reduced activation in left inferior frontal and temporo-parietal regions but increased activation of medial frontal regions, which may potentially be related to neurobiological mechanisms underlying cognitive and clinical phenotypes of OCD.

Autism spectrum disorder (ASD) is a neurodevelopmental disorder characterized by social and communication difficulties and stereotyped repetitive behaviors (1) with a prevalence of 0.6% to 2.0%, predominantly in male individuals (2). Obsessive-compulsive disorder (OCD) is characterized by recurrent, intrusive, and distressing thoughts (obsessions) and repetitive behaviors (compulsions) (1), affecting 1% to 3% of the population, with a higher prevalence in boys among pediatric samples (3). Rates of comorbidity of OCD in autistic children have been estimated to be as high as 37% (4). Conversely, estimates of ASD rates in OCD patients are lower at around 6% (5, 6). Clinically, compulsions in OCD are sometimes difficult to separate from repetitive behaviors in ASD. Both disorders also commonly present with inattention and even attention-deficit/hyperactivity disorder (ADHD), which may in some cases contribute to respective phenotypes including attention problems (7, 8). These overlaps have been attributed to shared genetic risk and biological mechanisms as well as diagnostic mislabeling (9), highlighting a need to improve understanding of the underlying neural mechanisms to disentangle comorbidity between the disorders and identify novel biomarkers and treatment targets (10).

Vigilance incorporates sustained attention, or the ability to maintain focus toward infrequently occurring stimuli (11), and focused attention, or the ability to concentrate on one stimulus while excluding the influence of other stimuli (12). There is evidence for deficits in vigilance and sustained attention in ASD (13, 14), albeit with some negative findings (15). In OCD, some studies support attention deficits across various domains (focused attention, sustained attention, selective attention, attention span, and information processing) relative to control subjects (16, 17, 18), whereas other studies find no deficits (19, 20). However, focused attention is perhaps the most widely studied attention domain in OCD, and the majority of studies support focused attention deficits (12). Attentional priority to obsessions is a key feature of OCD, and individuals with OCD have shown self-reported impaired attentional control (21). Thus, impairments in focused and sustained attention seemingly fit with clinical characteristics of the disorder and have been supported by the neuropsychological literature (12). Discrepancy is likely due to heterogeneous samples and tasks.

On cognitive and symptom-based measures, ASD has been related to inattention. Thus, ASD can be characterized by short attention span, and impulsivity and inattention symptoms are common (22). Furthermore, individuals with ASD are typically impaired on neurocognitive measures of sustained and selective attention (7). There is evidence for fronto-striatal, parietal, and cerebellar abnormalities in ASD during selective and flexible attention (23, 24). Specifically, hypoactivity has been observed in ASD in middle–frontal gyrus, caudate, and anterior cingulate cortex (ACC) (25). However, only two functional magnetic resonance imaging (fMRI) studies have measured sustained attention in ASD: one in adolescents (26) and one in a combined sample of adolescents and adults (13). These investigations found that individuals with ASD exhibited decreased activation in left dorsolateral–prefrontal striato-thalamic and parietal regions but increased activation in the cerebellum, presumably compensating for frontal hypoactivation, and in precuneus, reflecting poor deactivation of the default mode network linked to increased mind wandering (26). The first cross-sectional fMRI developmental investigation of sustained attention in ASD found that control subjects, but not individuals with ASD, had enhanced activation in inferior and dorsolateral–prefrontal, striatal, temporal, and cerebellar regions with increasing age, suggesting abnormal functional maturation of attention networks in ASD (13).

Clinical symptoms of inattention have been reported especially in pediatric patients with OCD (27), and patients with OCD have shown deficits in selective and focused attention (12). Pediatric and adult studies of OCD across various cognitive domains have suggested that the disorder is characterized by dysfunctional attention networks involving basal ganglia and medial and orbitofronto-striatal regions (16). However, there is additional evidence that abnormalities may also be driven by dysfunctional temporo-parietal and cerebellar networks (28, 29), supporting phenotypes of distracted focused attention to external stimuli and inability to disengage from obsessions. Specifically, patients with OCD exhibit hypoactivation in lateral–prefrontal cortex (PFC), medial–orbitofrontal cortex, and caudate but increased activation in ventrolateral PFC and ACC during selective and other attention-based tasks (18, 30), suggesting top-down ventrolateral PFC and ACC control over striatal underactivation. However, no fMRI studies have examined vigilance in OCD or compared ASD and OCD.

Given diagnostic overlap and potential etiological links between ASD and OCD, it is critical to understand neurofunctional mechanisms that are shared or unique between these disorders. Work has begun to focus on delineating neural mechanisms between these disorders (31), but a comparison in the context of attention is lacking. Despite a dearth of robust neurocognitive associations between attention problems and these disorders, investigating this domain in ASD and OCD may be useful for pinpointing differences or similarities in associated brain networks giving rise to clinical phenotypes in each disorder. Thus, this study compared brain function of boys with ASD, boys with OCD, and typically developing control boys during a parametrically modulated fMRI vigilance task with increasing sustained attention loads. fMRI investigations of psychomotor vigilance using other paradigms (e.g., continuous performance test) in healthy adolescents and adults showed activation in inferior and dorsolateral–prefrontal, striato-thalamic, parieto-temporal, and cerebellar regions (32, 33). Therefore, we hypothesized that both disorders would show underactivation in inferior frontal and dorsolateral–prefrontal striato-cerebellar sustained attention networks relative to control boys and that this effect would be more pronounced with increasing attention load.

## Methods and Materials

### Participants

A total of 60 right-handed (34) boys [20 typically developing control boys, 20 boys with ASD, and 20 boys with OCD, 11–17 years old, IQ ≥ 70 (35)] were included. ASD diagnosis was made by a psychiatrist using ICD-10 criteria (36) and confirmed with the Autism Diagnostic Interview–Revised (37). The Autism Diagnostic Observation Schedule (38) was also completed. All boys with ASD reached cutoffs for autism in all domains of the Autism Diagnostic Interview–Revised and the Autism Diagnostic Observation Schedule. Based on the structured interview, comorbidity with other disorders including OCD was excluded by a consultant psychiatrist. Parents also completed the Social Communication Questionnaire (39) and the Strengths and Difficulties Questionnaire (SDQ) (40) (see Supplement). Participants with ASD had a physical examination to exclude medical disorders and biochemical, hematological, and chromosomal abnormalities associated with ASD. All boys with ASD were medication naïve.

The 20 boys with OCD were recruited from a national and specialist OCD clinic at the Maudsley Hospital (London, UK). OCD diagnosis was made by a psychiatrist or clinical psychologist in accordance with ICD-10 criteria after an in-depth, semistructured interview between patient and clinician was used to administer an expanded version of the Children’s Yale–Brown Obsessive Compulsive Scale (CY-BOCS) (41). Absence of comorbidity, including ASD, was confirmed by a consultant psychiatrist after administration of the structured CY-BOCS interview. Parents completed the SDQ. Four boys with OCD were prescribed stable doses of selective serotonin reuptake inhibitors (see Supplement).

The 20 healthy age- and handedness-matched control boys were recruited by advertisement and initially screened over the phone for the current or lifetime presence of any exclusion criteria including comorbidity. Healthy control boys scored below clinical cutoffs on the SDQ and the Social Communication Questionnaire and had no history of any psychiatric or physical comorbidity.

Exclusion criteria included comorbid psychiatric disorders, medical disorders affecting brain development, drug or alcohol dependency, head injury, genetic conditions associated with ASD, abnormal brain structural MRI findings, and MRI contraindications. A total of 31 individuals (15 control and 16 ASD) also participated in our fMRI studies of sustained attention in ASD versus ADHD (26) and functional maturation of sustained attention networks in ASD versus control individuals (13). Some participants participated in other fMRI tasks during their visit, data from which are published elsewhere (42, 43, 44, 45).

The study was conducted in accordance with the Declaration of Helsinki. Ethical approval was obtained from the local research ethics committee (05/Q0706/275). Study details were explained to children and guardians, and written informed consent was obtained for all participants.

### Psychomotor Vigilance Task

Subjects practiced the task briefly in a mock scanner. The 12-minute task (13) is an adapted variant of psychomotor vigilance and delay tasks (46, 47) requiring sustained and focused attention. Subjects responded as quickly as possible within 1 second via a right-handed button press on presentation of a timer counting up in milliseconds from zero. A premature response was recorded if the button was pressed before timer presentation. The timer appeared after short predictable delays of 0.5 second in series of 3 to 5 stimuli (260 total), or after an unpredictable delay of 2, 5, or 8 seconds (20/each), pseudorandomly interspersed into blocks after 3 to 5 delays of 0.5 second. The 0.5-second delays are typically anticipated, placing a larger demand on sensorimotor synchronization (48), whereas the longer infrequent delays place a higher load on sustained attention/vigilance.

### Analysis of Performance

Univariate repeated-measures analyses of variance (ANOVAs) with group as a between-subject factor and delay (2, 5, or 8 seconds) as a within-subject repeated measure examined group differences and delay effects on mean reaction time (MRT), intrasubject response variability (standard deviation) of reaction time (SD_intrasubject_), and omission errors.

### fMRI Acquisition

Gradient-echo echo planar magnetic resonance imaging data were acquired on a 3T General Electric Signa HDx Twinspeed scanner (Milwaukee, WI) using a quadrature birdcage head coil. In each of 22 noncontiguous planes parallel to the anterior–posterior commissure, 480 T2*-weighted images depicting blood oxygenation level–dependent (BOLD) contrast spanning the whole brain were acquired (echo time = 30 ms; repetition time = 1.5 seconds; flip angle = 60^o^; in-plane resolution = 3.75 mm; slice thickness = 5.0 mm; slice skip = 0.5 mm). A whole-brain high-resolution structural scan (inversion recovery gradient echo planar imaging) on which to superimpose the activation maps was also acquired in the intercommissural plane (echo time = 40 ms; repetition time = 3 seconds; flip angle = 90^o^; slices = 43; slice thickness = 3.0 mm; slice skip = 0.3 mm) providing complete brain coverage.

### fMRI Analysis

Event-related activation data were acquired in randomized trial presentation and analyzed using nonparametric methods [XBAM v4.1, www.brainmap.co.uk (49, 50)]. XBAM makes no normality assumptions, uses median statistics to control outlier effects, and uses permutation testing, giving excellent type I error control (51). After preprocessing, time-series analysis for individual subjects was based on published wavelet-based data resampling methods for fMRI data (see Supplement) (50, 52).

For between-group comparisons, a 3 × 3 split-plot ANOVA (3 delays and 3 groups) was tested for group, delay, and group by delay interaction effects using a randomization-based test for voxel or cluster-wise differences (50). Less than 1 false positive activation cluster was expected at *p* < .05 (voxel level) and *p* < .01 (cluster level). Statistical measures of BOLD response for each participant were then extracted in each significant cluster, and post hoc *t* tests were conducted to identify between-group differences.

### Influence of Behavior, Symptoms, and Medication

To examine whether activation in regions showing a group by delay interaction was related to clinical symptoms or task performance, we extracted statistical BOLD responses for the longest delay (the delay with the largest group effect) from these clusters and correlated this (Spearman two tailed) with MRT and SD_intrasubject_ within each group. Within diagnostic groups, we correlated BOLD responses from clusters that were abnormal relative to control boys (e.g., cerebellum/occipital in both groups and the other three clusters in OCD; see Results) with disorder-relevant symptom measures, Autism Diagnostic Observation Schedule social/communication subscales for ASD and CY-BOCS scores for OCD.

To test for medication effects on activation for the 4 boys with OCD who were prescribed selective serotonin reuptake inhibitors, analyses were repeated covarying for medication status and excluding medicated participants.

## Results

### Participants

Groups did not differ on age or IQ (Table 1). As expected, groups differed on total scores and subscores of the SDQ. Post hoc tests showed that on total and peer relations subscales, all patients were impaired relative to healthy control boys, but boys with ASD were more severely impaired than boys with OCD (total: all *p*s < .001; peer: all *p*s < .05). On emotional subscales, both diagnostic groups were impaired relative to control boys (*p* < .001) but did not differ from each other. On prosocial and hyperactivity/inattention subscales, boys with ASD were impaired relative to control boys and boys with OCD (*p* < .001), who did not differ from control boys. On the conduct problems subscale, only boys with ASD differed from control boys (*p* < .005).

**Table 1:** Participant Characteristics for Healthy Control Boys and Boys with ASD or OCD

Variable	HC (*n* = 20), Mean (SD)	ASD (*n* = 20), Mean (SD)	OCD (*n* = 20), Mean (SD)	*F* Test	Degrees of Freedom	*p* Value
Age, Years	15.1 (2.0)	15.2 (1.3)	15.7 (1.4)	0.9	2,57	.43
IQ	119.7 (11.9)	112.2 (14.4)	117.7 (13.4)	1.7	2,57	.19
SCQ Total Score	2.32 (2.3)	18.66 (8.1)	–	77.0	1,47	<.001
SDQ Total Score	5.6 (4.2)	19.7 (6.8)	12.5 (5.6)	35.6	2,66	<.001
SDQ Emotional Distress Subscale	0.9 (1.8)	4.4 (2.9)	4.4 (2.6)	13.1	2,66	<.001
SDQ Conduct Subscale	0.9 (1.1)	2.7 (2.2)	1.9 (1.5)	6.6	2,66	.003
SDQ Peer Relations Subscale	1.5 (1.7)	6.6 (2.3)	3.3 (3.0)	28.7	2,66	<.001
SDQ Hyperactive Impulsive/Inattentive Subscale	2.7 (2.4)	5.9 (2.6)	3.0 (2.7)	12.5	2,66	<.001
SDQ Prosocial Behavior Subscale	8.4 (2.4)	4.4 (2.4)	7.7 (2.6)	18.6	2,6	<.001
ADOS Communication Score	–	3.6 (1.2)	–	–	–	–
ADOS Social Interaction Score	–	9.0 (2.3)	–	–	–	–
ADOS Communication + Social	–	12.7 (3.1)	–	–	–	–
ADOS Stereotypy Score	–	1.5 (1.5)	–	–	–	–
ADI Communication Score	–	16.6 (4.7)	–	–	–	–
ADI Social Interaction Score	–	20.0 (5.3)	–	–	–	–
ADI Repetitive Behavior Score	–	6.5 (2.4)	–	–	–	–
CY-BOCS Total Score	–	–	22.3 (5.8)	–	–	–
CY-BOCS–Obsessions	–	–	10.8 (3.6)	–	–	–
CY-BOCS–Compulsions	–	–	12.0 (3.1)	–	–	–

ADI, Autism Diagnostic Interview; ADOS, Autism Diagnostic Observation Schedule; ASD, autism spectrum disorder; CY-BOCS, Children’s Yale–Brown Obsessive Compulsive Scale; HC, healthy control; OCD, obsessive-compulsive disorder; SCQ, Social Communication Questionnaire; SDQ, Strengths and Difficulties Questionnaire.

### Performance Data

Repeated-measures ANOVAs showed no significant within-subjects effect of delay on MRT, *F*_1.7,__95.1_ = 1.99, *p* = .15, SD_intrasubject_, *F*_2,114_ = 0.56, *p* = .57, or omissions, *F*_1.7,98.8_ = 0.48, *p* = .59.

There was no significant group effect on MRT, *F*_2,57_ = 1.50, *p* = .23, SD_intrasubject_, *F*_2,57_ = 0.78, *p* = .46, or omissions, *F*_2,57_ = 1.00, *p* = .37.

There was no significant group by delay interaction effect for MRT, *F*_3.3,95.1_ = 0.77, *p* = .53, SD_intrasubject_, *F*_4,114_ = 1.71, *p* = .15, or omissions, *F*_3.5,98.8_ = 1.82, *p* = .14 (Supplemental Table S1).

### Movement

Groups did not differ on minimum (*F*_2,57_ = 1.00, *p* = .38), maximum (*F*_2,57_ = 0.30, *p* = .76), or mean (*F*_2,57_ = 0.003, *p* = 1.00) head translation in three-dimensional Euclidian space.

### Group Maps of Brain Activation

Images of within-group brain activation for each delay (2, 5, or 8 seconds) contrasted against 0.5-second trials are described in Supplemental Figure S1.

### Delay Effect

All subjects showed distributed activation with increasing delay in a bilateral network comprising ventromedial/dorsolateral/inferior PFC, anterior/posterior cingulate, basal ganglia supplementary motor area, temporo-parietal and cerebellar regions, and thalamus and hippocampal gyri (Supplemental Figure S2).

### Group Effect

**Figure 1:**
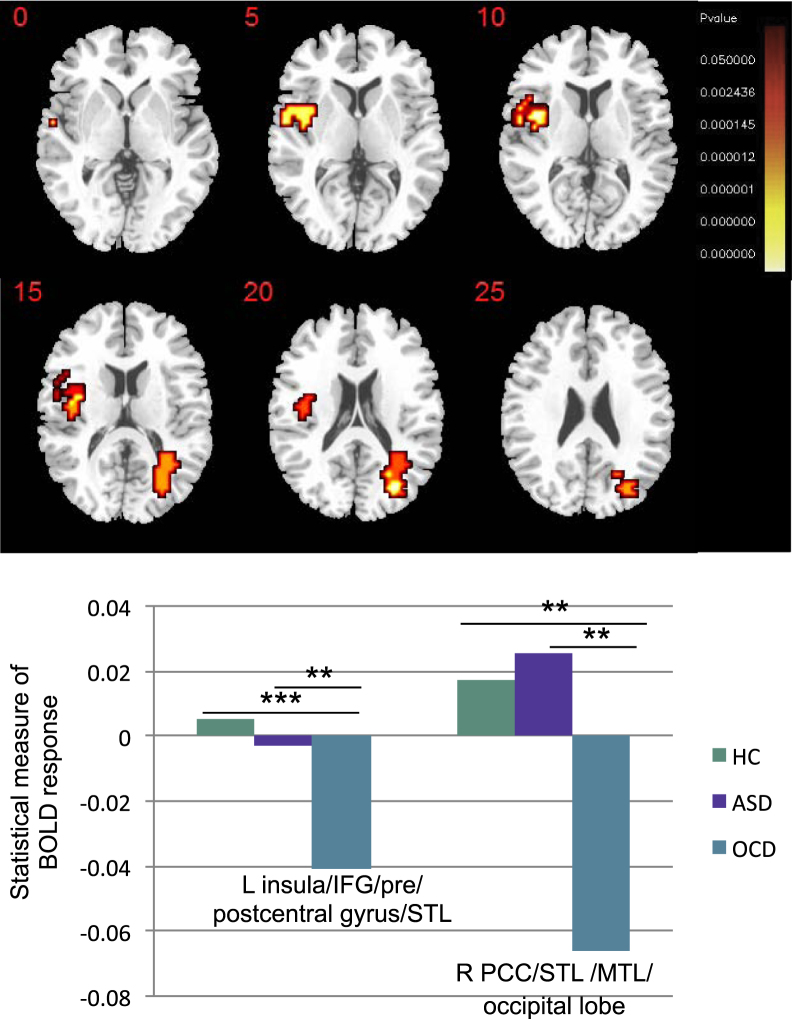
Between-group differences in brain activation among healthy control (HC) boys, boys with autism spectrum disorder (ASD), and boys with obsessive-compulsive disorder (OCD). Analysis of variance shows the main effect of group on brain activation for all delays (2, 5, and 8 seconds) combined, contrasted against 0.5-second trials. Talairach *z* coordinates are shown for slice distance (in mm) from the intercommissural line. The right side corresponds with the right side of the brain. **p < .005, ***p < .001. BOLD, blood oxygen--level dependent; IFG, inferior frontal gyrus; L, left; MTL, middle temporal lobe; PCC, posterior cingulate cortex; R, right; STL, superior temporal lobe.

Split-plot ANOVA revealed significant group effects in left insula/inferior frontal gyrus (IFG) extending into pre/postcentral gyrus/superior temporal lobe (STL) and right posterior cingulate cortex/STL extending into middle temporal lobe/occipital lobe (Figure 1 and Table 2).

**Table 2:** ANOVA Effects for Brain Activation Differences Among Boys With ASD, Boys With OCD, and Healthy Control Boys

Subject Contrast	Brain Regions of Activation	Brodmann Areas	Peak Talairach Coordinates, *x, y, z*	Voxels	*p* Value
Main Effect of Group					
OCD < HC, ASD	L insula^a^/IFG/pre/postcentral gyrus/STL	45/44/6/4/43/22	−40, 0, −2	49	.009
OCD < HC, ASD	R PCC^a^/STL^a^/MTL/occipital lobe	23/31/22/39/19	29, −63, 9	38	.006
Group by Delay Interaction Effects					
OCD < HC, ASD	L insula^a^/IFG/precentral gyrus/STL/MTL	47/44/45/6/41/22/21	−43, 11, −2	91	.0008
OCD < HC, ASD	L IPL^a^/pre/postcentral gyrus	40/6/4/3/1	−51, −30, 37	48	.0009
OCD > ASD, HC	rmPFC^a^/superior frontal/ACC	9/10/32	11, 56, 20	63	.001
ASD, OCD > HC	Cerebellum vermis^a^/occipital lobe/lingual gyrus	17/18/19	7, −70, −13	49	.003

ACC, anterior cingulate cortex; ANOVA, analysis of variance; ASD, autism spectrum disorder; HC, healthy control; IFG, inferior frontal gyrus; IPL, inferior parietal lobe; L, left; MTL, middle temporal lobe; OCD, obsessive–compulsive disorder; PCC, posterior cingulate cortex; R, right; rmPFC, rostromedial prefrontal cortex; STL, superior temporal lobe.

a Indicates cluster peak.

Post hoc analyses showed that boys with OCD had decreased activation in left insula/IFG relative to control boys (*p* < .001) and boys with ASD (*p* = .002), who did not differ from control boys, and in right posterior cingulate cortex/STL relative to control boys (*p* = .002) and boys with ASD (*p* = .001), who did not differ from control boys.

### Group by Delay Interaction Effects

**Figure 2:**
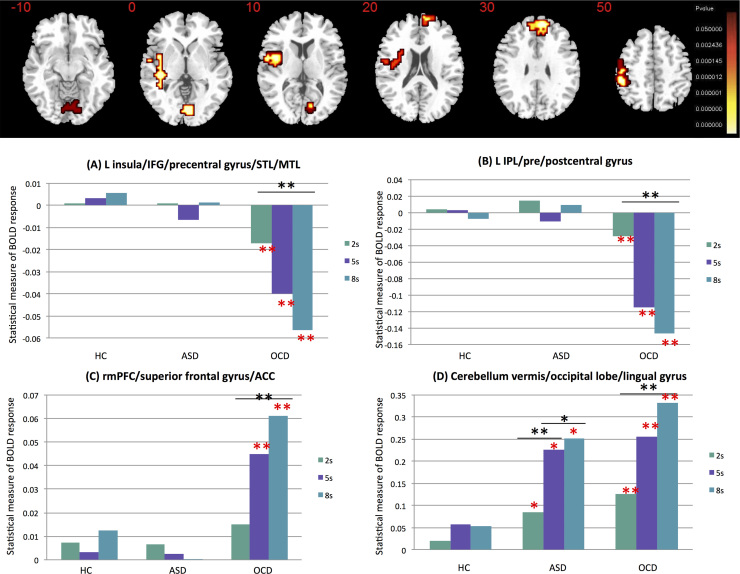
Group by delay interaction among healthy control boys, boys with autism spectrum disorder (ASD), and boys with obsessive-compulsive disorder (OCD) and delay condition (2, 5, or 8 seconds). Analysis of variance shows group by delay interaction effects on brain activation. Talairach *z* coordinates are shown for slice distance (in mm) from the intercommissural line. The right side corresponds with the right side of the brain. Red asterisks indicate significant difference between diagnostic group and control boys. Black asterisks indicate significant difference within group between conditions. *p < .05, **p < .005. ACC, anterior cingulate cortex; BOLD, blood oxygen--level dependent; IFG, inferior frontal gyrus; IPL, inferior parietal lobe; L, left; MTL, middle temporal lobe; rmPFC, rostromedial prefrontal cortex; STL, superior temporal lobe.

Split-plot ANOVA showed significant group by delay interaction effects in four clusters, one of which overlapped with observed results in the group effect analysis: left insula/IFG extending into precentral gyrus/STL/middle temporal lobe, left inferior parietal lobe/pre/postcentral gyrus, rostromedial PFC/superior frontal gyrus/ACC, and cerebellar vermis/occipital lobe/lingual gyrus (Figure 2 and Table 2).

Post hoc analyses showed that in left IFG/insula (Figure 2A) and left inferior parietal lobe/pre/post-central gyrus (Figure 2B), boys with OCD had progressively reduced activation with increasing delay (*p* < .005) relative to boys with ASD and control boys (*p* < .005), who did not differ and whose activation in this region did not change with delay. In rostromedial PFC (Figure 2C), boys with OCD had increased activation with increasing delay (*p* < .004). There was no between-group difference in the 2-second condition, but for 5 and 8 seconds, boys with OCD had increased activation relative to boys with ASD and control boys (*p* < .005), who did not differ and whose activation in this region did not change with delay. In cerebellum/occipital lobe (Figure 2D), diagnostic groups had increased activation with increasing delay (ASD: *p* < .05; OCD: *p* < .005) and shared enhanced activation in all delays relative to control boys (ASD vs. control: *p* < .04; OCD vs. control: *p* < .001).

### Influence of Performance, Clinical Symptoms, and Medication

Within patients, there were no significant correlations between clinical measures and brain activation. There was no relationship between performance and activation within any of the three groups.

When medication was covaried, results remained unchanged, suggesting that medication did not significantly affect activation differences observed during the task. When analyses were repeated excluding these 4 patients, findings were still observed at a more lenient significant threshold of *p* < .05, likely as an effect of reduced power to detect group differences.

## Discussion

This is the first fMRI study to directly compare boys with ASD and boys with OCD to investigate shared or disorder-specific abnormalities in brain function and is the first to make this comparison using sustained attention. During a parametrically modulated vigilance task, boys with OCD had disorder-specific patterns of reduced activation in left-lateral inferior fronto-parieto-temporal regions but enhanced activation in medial frontal regions with increasing task difficulty relative to healthy control boys and boys with ASD, who did not differ from one another. Both disorders shared enhanced activation relative to control boys with increasing delay in cerebellum/occipital lobe.

Neither diagnostic group differed from control boys on task performance (MRT, SD_intrasubject_). Across all groups, participants activated distributed ventromedial, dorsolateral, and inferior prefronto-striato thalamic and temporo-parietal networks with increasing attention load, suggesting that the task elicited the expected brain response given that dorsolateral and inferior fronto-striato-temporo-parietal networks are important for maintaining attention (26, 33).

Boys with OCD had disorder-specific activation decreases relative to control boys and boys with ASD in left insula, IFG, and STL, which furthermore showed decreases in activation as a function of increasing attention load in the OCD group but not in the other groups. The insula is involved in salience detection and timing functions (53, 54). Reduced insular and paralimbic activation presumably reflects these regions’ role in motivation control, which may influence attention (55). Thus, OCD-specific deactivation in this region may be a disorder-specific signature, shifting cognitive resources away from internal thoughts to elicit task-relevant attention comparable to control boys. The insula is furthermore involved in switching between task-related central executive networks and task-unrelated default mode network activations, suggesting that the insula facilitates dampening down of default mode activity during sustained attention (56). Thus, OCD-specific decreased insula activation with increasing attention load could relate clinically to difficulty maintaining attention toward task-relevant stimuli due to attentional priority to task-unrelated internally generated obsessions.

Left IFG is a key part of the ventral attention network and, along with lateral temporo-parietal regions, is important for attention-orienting maintenance (57). Investigations of ventral sustained attention systems generally implicate right-hemispheric regions (56, 58) [but see (59) for evidence of left-hemispheric activation]. It is conceivable that observed left-hemispheric activation reflects sensorimotor effects of the right-handed button press. This particular vigilance task has shown in previous studies (13, 26) to elicit predominantly left-lateralized activation in fronto-insular regions in healthy adults and children, supported by our within-group findings of predominantly left hemispheric fronto-insular activation presented in the Supplement. Moreover, this effect could be due to this region’s role in motor timing and sensorimotor synchronization (54). There have been age-related findings of increased IFG/insula activation during sustained attention and cognitive control between childhood and adulthood (32, 60), suggesting abnormal functional maturation in OCD relative to ASD and control subjects, in line with structural MRI studies showing abnormal white matter development (61) and decreased cortical thickness in adults with OCD relative to control adults (62). Taken together, this evidence suggests that abnormal inferior frontal functional maturation may be a potential biomarker for OCD.

Boys with OCD also had disorder-specific decreased activation in inferior parietal and superior and middle temporal regions with increasing attention load relative to control boys and boys with ASD. Inferior parietal/superior temporal lobes show decreased activation as a function of time during vigilance in healthy individuals (63), suggesting that this effect may be exaggerated in OCD, particularly because the effect was more pronounced with increasing delays. Similar reductions in inferior fronto-parieto-cerebellar vigilance and motivation networks have been found during sustained attention in adolescents with ADHD (26, 64, 65), suggesting that abnormalities may represent underlying mechanisms of inattention that are disorder specific to OCD versus ASD. Interestingly, our previous study comparing vigilance in ASD and ADHD (26) found reduced left dorsolateral–prefrontal activation in younger subjects with ASD relative to control subjects, but these findings were based on a comparison of participants with ASD and participants with ADHD not included in the current study, potentially reflecting age-related differences.

Inferior parietal and temporal regions are involved in attentional orienting to time and readjustment of attention after disengagement (58). Thus, reduced activation in this region with increasing delay in OCD but not ASD could suggest that sustained attention is more neurofunctionally impaired in OCD than in ASD, particularly during increasing attention load. This may be related clinically to a poor ability to reengage with task-relevant attention in individuals with OCD if they become distracted by intrusive thoughts.

Whereas participants with OCD showed disorder-specific patterns of progressively decreased activation with increasing attention load in lateral fronto-parieto-temporal regions, they also showed progressively increased activation in medial frontal ACC/medial PFC (MPFC) relative to boys with ASD and control boys. MPFC/ACC hyperactivation is a classic pattern in OCD during attention-based tasks (28), particularly in the context of error monitoring (29). Anterior MPFC has been implicated in withholding preplanned responses and seems to facilitate action intention across delays (66). Moreover, this region may downregulate motor activity, acting as a control mechanism inhibiting response until target presentation (67). Thus, increased MPFC activation in OCD with increasing attention load as left lateral fronto-temporo-parietal activation decreased could reflect compensation to elicit behavior similar to control boys and boys with ASD.

Taken together, OCD-specific findings of reduced left inferior frontal and temporo-parietal activation but increased MPFC/ACC activation relative to control subjects is in line with common patterns of reduced lateral fronto-temporo-parietal activation/morphology in OCD but increased function/morphology in MPFC during inhibition, error monitoring, and symptom provocation (31, 68, 69). The current findings extend this evidence to attention and vigilance, suggesting that this pattern may be more characteristic of OCD pathophysiology.

Both disorders shared increased activation in cerebellar vermis/lingual gyrus, with increasing delays relative to control subjects. The cerebellum is implicated in the pathophysiology of ASD (70) and OCD (71). In ASD, this fits with enhanced cerebellar vermis activation relative to control subjects and subjects with ADHD during sustained attention (26) and may be associated with structural deficits (72) and abnormal fronto-cerebellar connectivity in ASD (73). Moreover, the cerebellum has been implicated in attention to time intervals (74), suggesting that ASD and OCD share deficits in this aspect of attention orienting involved in vigilance to temporal delay. A recent fMRI meta-analysis of sustained attention (56) found that the cerebellar vermis is activated with increasing delays, suggesting its role in timing and anticipation of motor responses, in line with findings of impaired anticipatory timing in patients with cerebellar lesion (75). Abnormalities in the lingual gyrus have been linked to impaired sustained attention in depression (76). The current results extend this finding to OCD, suggesting that posterior regions are implicated in circuitry relevant to vigilance/attention and impaired in clinical populations associated with internal thought and rumination. The finding of progressively increased activation in this region in boys with OCD relative to control boys may compensate for neurofunctional impairments in OCD in left lateral inferior fronto-temporo-parietal attention-related regions, leading to preserved task performance in this group.

Interestingly, while neither disorder showed performance deficits, there were shared and disorder-specific neurofunctional abnormalities for OCD relative to ASD. There are several explanations for this. Subject numbers required for fMRI studies are smaller than those required for neuropsychological analyses, reducing statistical power for behavioral analysis. Moreover, the aim of fMRI studies is to understand differences in neural networks between cases and controls during task performance. To relate activation differences to pathology and not simply to performance differences, it is important that performance did not differ between groups (77). Across child and adult psychiatry, neurofunctional differences have been demonstrated between cases and controls despite comparable task performance (78, 79, 80, 81). Therefore, apparently similar task performance is achieved with different neural activation between groups, particularly in boys with OCD, who showed disorder-specific patterns of decreased lateral inferior fronto-temporal and increased medial frontal activation. It is possible that the increased medial frontal activation may be compensatory in response to reduced lateral inferior fronto-temporal activation, suggesting that patients with OCD relied less on lateral and posterior attention mechanisms and more on medial prefrontal regions for task performance. Conversely, both disorders achieved comparable performance to control subjects with increased cerebellar-occipital activation, which may reflect shared neurofunctional mechanisms of enhanced default mode activity.

Despite these differences in brain activation, groups did not differ in performance. However, this is an advantage because brain activation was therefore not confounded by performance differences. Brain activation is typically more sensitive than performance to detect group differences in these patient groups [see, e.g., (44, 82, 83, 84, 85, 86)]. There was furthermore no correlation between clinical measures or task performance and activation. Whereas the subject numbers have been shown to be sufficient for fMRI analyses (51), the performance and correlation analyses are underpowered, which may explain the negative findings.

This study has several limitations. While patients with psychiatric comorbidities were excluded, we cannot rule out the presence of subthreshold symptoms of other disorders such as ADHD. This is in line with the debate around comorbidity versus overlapping phenotypes and their respective contribution to behavior and clinical presentation, particularly in the context of ASD and ADHD (22). It would have been interesting to investigate correlations with more detailed attention-based behavioral questionnaires. Nevertheless, SDQ scores have been shown to strongly correlate with inattention symptoms on other measures such as the Child Behavior Checklist (40, 87). Similarly, a standard OCD measure (e.g., CY-BOCS) was not administered to patients with ASD. However, absence of OCD comorbidity in individuals with ASD was confirmed by a psychiatrist based on a structured interview. A study strength is the inclusion of noncomorbid, medication-free boys with ASD. However, 4 boys with OCD were prescribed selective serotonin reuptake inhibitors. There is evidence for neurofunctional effects of serotonin (88), but after covarying for and excluding medicated patients, the findings remained (albeit at a slightly more lenient threshold), suggesting that medication did not significantly affect brain function. Lastly, phenotypes of OCD are closely linked to anxiety (89). Whereas anxiety ratings were not collected before scanning, the possibility that OCD patients were more anxious compared with the other groups should not be discounted. This may partially explain the OCD group’s reduced recruitment of attention-related brain regions and suggests that activation differences are indicative of anxiety as opposed to fundamental attention problems.

Future work could compare ASD individuals with and without comorbid OCD with noncomorbid OCD individuals, building on this novel comparison to elucidate the mechanisms underlying clinical overlap of ASD and OCD. Moreover, it would be interesting to compare these patient groups with attention-related disorders such as ADHD to provide further insight into shared and/or disorder-specific neurofunctional attention mechanisms.

### Conclusions

This study provides the first evidence suggesting that adolescents with OCD have disorder-specific abnormalities in sustained attention networks, including left inferior and medial PFC and temporo-parietal regions, relative to adolescents with ASD, who had no frontal abnormalities. Findings suggest lateral inferior/medial fronto-temporo-parietal abnormalities during sustained attention may be a distinct neural signature of OCD but not of ASD. Individuals with ASD and OCD, however, shared abnormally enhanced activation in cerebellum/occipital lobe relative to healthy control individuals. These results provide promising evidence for identification of biomarkers that may clarify underlying mechanisms driving sustained attention and respective symptom profiles in autism and OCD.
