# Supplementary material for: Disorder-Specific and Shared Brain Abnormalities During Vigilance in Autism and Obsessive-Compulsive Disorder
Source: Biol Psychiatry Cogn Neurosci Neuroimaging. 2017 Nov;2(8):644–54. doi: 10.1016/j.bpsc.2016.12.005 (PMC5685008; doi:10.1016/j.bpsc.2016.12.005)
Supplement: Supplementary file 1 — Supplementary material [file mmc1.pdf]

Disorder-Specific and Shared Brain Abnormalities During Vigilance in Autism and Obsessive-Compulsive Disorder

Supplemental Information

SUPPLEMENTAL METHODS

Participants

All but 3 ASD participants scored above clinical threshold for ASD on the Social Communication Questionnaire (SCQ; (1)), but these patients were included on the basis of clinician-confirmed ASD diagnosis. Six ASD participants also scored above threshold for inattention/hyperactivity symptoms on the Strengths and Difficulties Questionnaire (SDQ; (2)) but were not excluded on the basis that attention problems are common in ASD and clinician confirmation that ASD symptoms were the sole/primary clinical concern for these patients.

One OCD patient scored above clinical cut-off for inattention/hyperactivity symptoms on the SDQ but was not excluded on the basis that communication and attention difficulties can be misconstrued for OCD-related symptoms and the fact that no OCD patients met criteria for ASD or ADHD based on clinical interview.

OCD Patient Medication Status

Patient 1: Sertraline 75mg

Patient 2: Sertraline 100mg

Patient 3: Sertraline 200mg

Patient 4: Fluvoxamine 100mg; risperidone 0.5mg

fMRI Data Analysis Methods

Individual analysis

Data were first processed to minimize motion-related artefacts (3). A 3-D volume consisting of the average intensity at each voxel over the entire experiment was calculated and used as a template. The 3D image volume at each time point was then realigned to this template by computing the combination of rotations (around the *x*, *y* and *z* axes) and translations (in *x*, *y* and *z*) that maximized the correlation between the image intensities and the volume in question and the template (rigid-body registration). Following realignment, data were then smoothed using a Gaussian filter (full-width at half-maximum (FWHM) 7.2 mm) to improve the signal-to-noise ratio of the images (3). Following motion correction, global detrending and spin-excitation history correction, time series analysis for each subject was conducted based on a previously published wavelet-based resampling method for fMRI data (4,5). At the individual-subject level, a standard general linear modeling approach was used to obtain estimates of the response size (beta) to each of the task conditions (2, 5 and 8s long delays) against an implicit baseline of 0.5s delays. We first convolved the main experimental conditions with 2 Poisson model functions (peaking at 4 and 8s). We then calculated the weighted sum of these 2 convolutions that gave the best fit (least-squares) to the time series at each voxel. A goodness-of-fit statistic (SSQ ratio) was then computed at each voxel consisting of the ration of the sum of squares of deviations from the mean intensity value due to the model (fitted time series) divided by that of the squares due to the residuals (original time series minus model time series). The appropriate null distribution for assessing significance of any given SSQ ratio was established using a wavelet-based data re-sampling method (5) and applying the model-fitting process to the resampled data. This process was repeated 20 times at each voxel and the data combined over all voxels, resulting in 20 null parametric maps of SSQ ratios for each subject, which were combined to give the

overall null distribution of SSQ ratio. This same permutation strategy was applied at each voxel to preserve spatial correlation structure in the data. Individual SSQ ratio maps were then transformed into standard space, first by rigid-body transformation of the fMRI data into a high-resolution inversion recovery image of the same subject, and then by affine transformation onto a Talairach template (6).

Group analysis

For the group-level analysis, less than 1 false positive-activated 3D cluster was expected at $p<0.05$ (voxel-level) and $p<0.01$ (cluster-level). A group-level activation map was produced for each group and each experimental condition (2, 5 and 8s) by calculating the median observed SSQ ratios at each voxel in standard space across all subjects and testing them against the null distribution of median SSQ ratios computed from the identically transformed wavelet-resampled data (5,7). The voxel-level threshold was first set to 0.05 and tests were conducted to identify voxels that might be plausibly activated followed by a test at a cluster-level threshold of $p<0.01$ to remove the false-positive clusters produced by the voxel-level test (4,5). Next, a cluster-level threshold was computed for the resulting 3D voxel clusters. The necessary combination of voxel and cluster level thresholds was not assumed from theory but rather was determined by direct permutation for each dataset, giving excellent type-II error control (4). Cluster mass rather than a cluster extent threshold was used to minimize discrimination against possible small, strongly responding foci of activation (4).

SUPPLEMENTAL RESULTS

Supplementary Table S1. Performance Data

Performance measure, mean (SD)	Delay	Controls (N=20)	ASD boys (N=20)	OCD boys (N=20)
MRT (ms)	0.5s	300.31 (42.56)	294.12 (54.25)	300.04 (30.40)
	2s	379.24 (37.16)	404.74 (44.32)	392.99 (43.55)
	5s	378.29 (45.01)	398.55 (46.69)	388.29 (48.09)
	8s	386.87 (50.78)	411.60 (54.95)	388.66 (45.38)
SDintrasubject	0.5s	65.68 (25.33)	85.11 (31.10)	77.06 (30.53)
	2s	67.08 (29.75)	75.52 (37.26)	69.09 (23.00)
	5s	54.59 (23.21)	68.35 (31.40)	77.07 (38.10)
	8s	65.92 (23.93)	70.22 (32.05)	64.28 (31.59)
Omissions (number)	0.5s	0.60 (1.63)	0.58 (1.61)	0.45 (1.00)
	2s	0.15 (0.67)	0.05 (0.23)	0.00 (0.00)
	5s	0.20 (0.89)	0.05 (0.23)	0.00 (0.00)
	8s	0.30 (1.13)	0.00 (0.00)	0.00 (0.00)

ASD-autism spectrum disorder; MRT-mean reaction time; ms-milliseconds; OCD-obsessive-compulsive disorder; s-seconds; SD-standard deviation

fMRI Data – Within-Group Activation Results

Within each group separately (controls, patients with ASD, patients with OCD), all groups showed distributed increased activation with increasing delay in a widespread network encompassing bilateral cerebellum and occipital lobe, medial and superior temporal regions, posterior cingulate cortex, pre and post-central gyrus, insula and predominately right dorsolateral prefrontal cortex extending into supplementary motor area as well as subcortical regions including bilateral thalamus and putamen. The OCD group (and controls to a lesser extent) also had increasing activation in dorsomedial prefrontal regions which was strongest in the 8s condition.

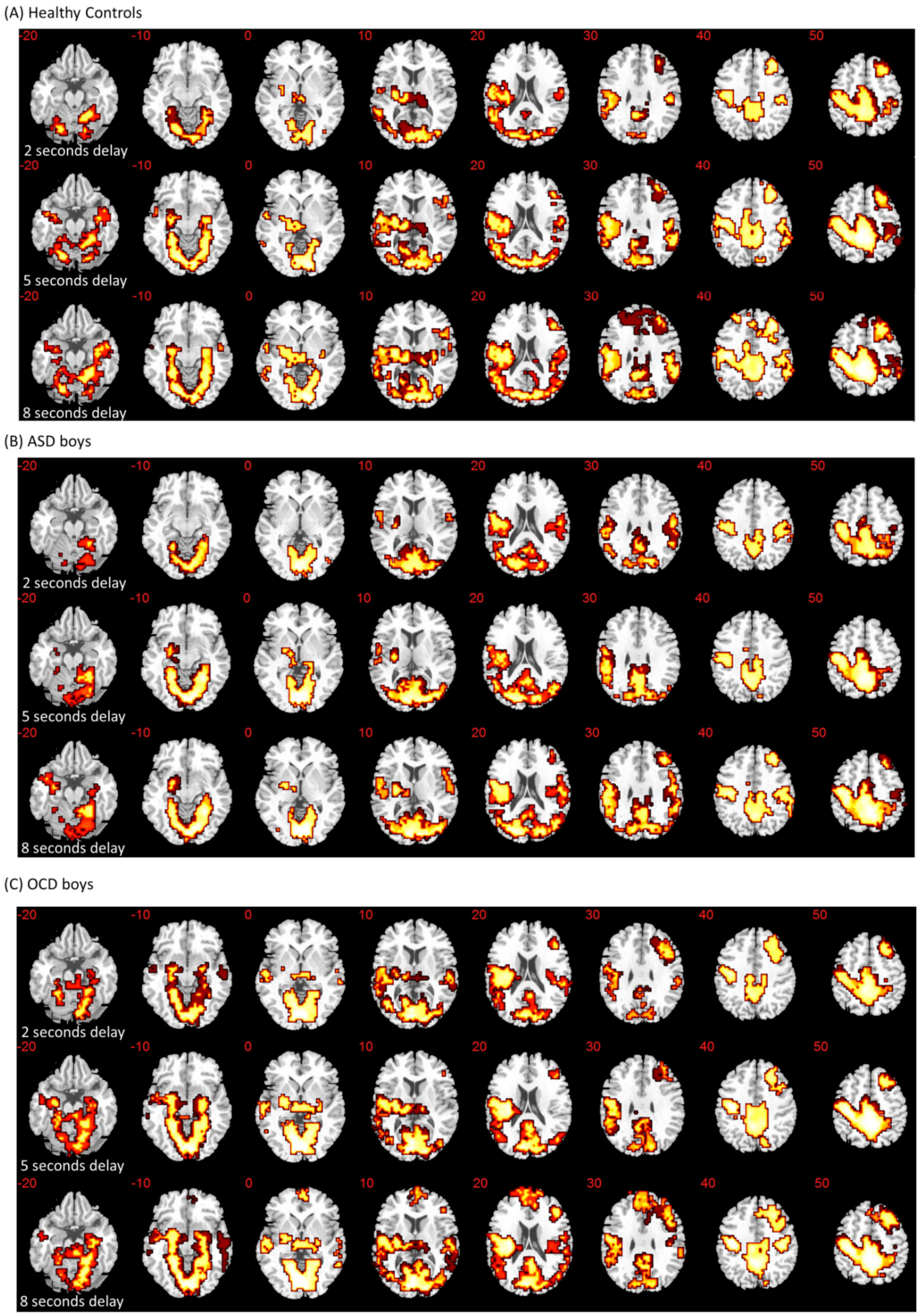

Supplementary Figure S1. Within-group brain activation for each delay condition (2, 5, 8s). Horizontal sections showing within-group brain activation for each delay condition of 2, 5 and 8 seconds for (A) healthy control boys, (B) boys with ASD and (C) boys with OCD. Talairach *z*-coordinates are shown for slice distance (in mm) from the intercommissural line. The right side of the image corresponds with the right side of the brain.

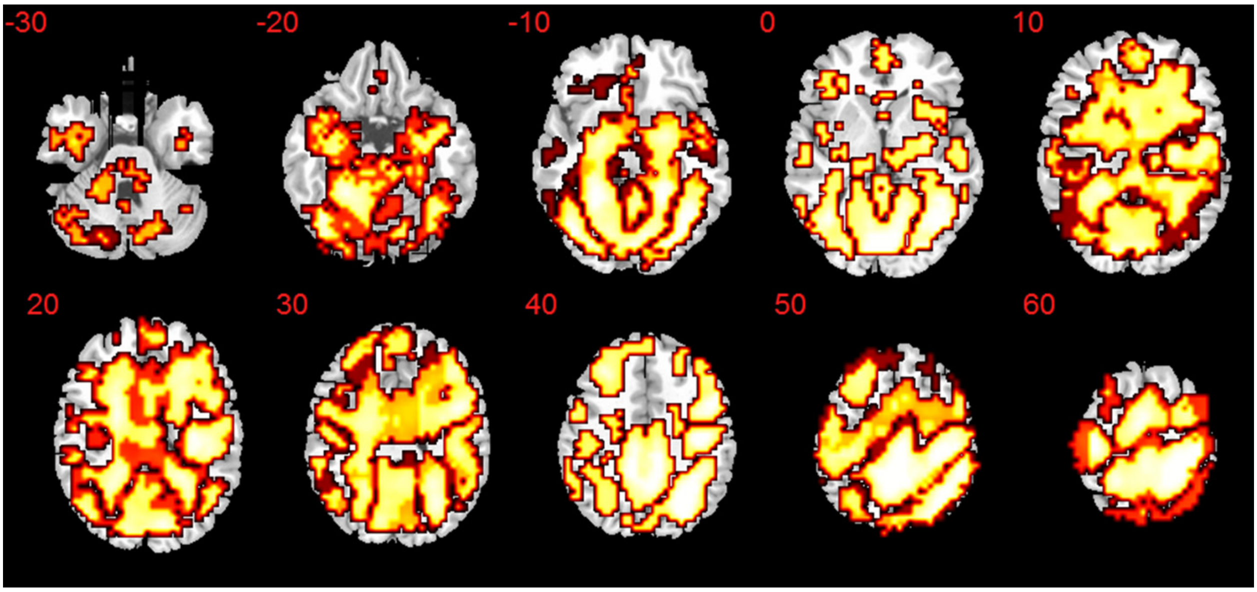

Supplementary Figure S2. Main effect of delay across healthy controls, boys with ASD and boys with OCD. Horizontal sections showing brain activation across all groups (healthy controls, boys with ASD and boys with OCD) with increasing delay (2, 5 and 8 seconds). Talairach *z*-coordinates are shown for slice distance (in mm) from the intercommissural line. The right side of the image corresponds with the right side of the brain.

SUPPLEMENTAL REFERENCES

1. Rutter M, Bailey A, Lord C (2003): *The social communication questionnaire: Manual*. Western Psychological Services.

2. Goodman R, Scott S (1999): Comparing the Strengths and Difficulties Questionnaire and the Child Behavior Checklist: Is Small Beautiful? *Journal of Abnormal Child Psychology*. 27:17-24.

3. Bullmore E, Brammer M, Rabe-Hesketh S, Curtis V, Morris R, Williams S, et al. (1999): Methods for diagnosis and treatment of stimulus-correlated motion in generic brain activation studies using fMRI. *Human Brain Mapping*. 7:38-48.

4. Bullmore ET, Suckling J, Overmeyer S, Rabe-Hesketh S, Taylor E, Brammer MJ (1999): Global, voxel, and cluster tests, by theory and permutation, for a difference between two groups of structural MR images of the brain. *Medical Imaging, IEEE Transactions on Medical Imaging* 18:32-42.

5. Bullmore E, Long C, Suckling J, Fadili J, Calvert G, Zelaya F, et al. (2001): Colored noise and computational inference in neurophysiological (fMRI) time series analysis: resampling methods in time and wavelet domains. *Human Brain Mapping*. 12:61-78.

6. Talairach J, Tournoux P (1988): *Coplanar stereotaxic atlas of the human brain, a 3-dimensional proportional system: an approach to cerebral imaging*. New York: Thieme.

7. Brammer MJ, Bullmore ET, Simmons A, Williams SCR, Grasby PM, Howard RJ, et al. (1997): Generic brain activation mapping in functional magnetic resonance imaging: A nonparametric approach. *Magnetic Resonance Imaging*. 15:763-770.
